# Efficacy of orbital radiotherapy in moderate-to-severe active graves’ orbitopathy including long-lasting disease: a retrospective analysis

**DOI:** 10.1186/s13014-020-01663-8

**Published:** 2020-09-21

**Authors:** Jin Hwa Choi, Jeong Kyu Lee

**Affiliations:** 1 Department of Radiation Oncology, Chung-Ang University College of Medicine, Seoul, Republic of Korea; 2 Department of Ophthalmology, Chung-Ang University College of Medicine, Seoul, Republic of Korea

**Keywords:** Graves’ orbitopathy, Radiotherapy, Timing, Predictive factors

## Abstract

**Background:**

We aimed to explore the efficacy of orbital radiotherapy (RT) in patients with moderate-to-severe active Graves’ orbitopathy (GO), including long-lasting disease, and to determine the predictive factors associated with treatment response.

**Methods:**

This was a retrospective study of 62 moderate-to-severe active GO patients treated with RT. Demographic data and ophthalmic findings prior to RT and at 3 and 6 months afterward were analyzed. Computed tomography was performed before and after RT to compare orbital volume change. We used logistic regression to determine the predictive factors for treatment response. Subjects were divided into early- and late-active phase groups based on GO duration of 24 months and treatment outcomes were compared with each other to observe the effects of RT timing on treatment response.

**Results:**

Forty (64.5%) and forty-six (74.1%) patients experienced improvements in GO at 3 and 6 months after radiotherapy, respectively. Ocular parameters such as clinical activity score (CAS), proptosis, extraocular muscle (EOM) limitation, and compressive optic neuropathy (CON) were improved by RT. Volumes of EOM significantly decreased after RT. The enlargement of EOMs and EOM limitation were predictive factors for a good response to RT. At 6 months after RT, 22 (68.8%) patients of late-active phase group exhibited improvement in GO, which is comparable to the number of 24 (80.0%) patients of early-active phase group. In the late-active phase group, CAS, diplopia, and visual acuity were improved significantly, but there was no change in EOM limitation.

**Conclusions:**

In moderate-to-severe active GO patients, orbital RT may help improve high CAS, proptosis, EOM limitation, and CON. The orbital RT in long-lasting active GO patients may be considered as treatments for the relief of symptoms including high CAS and poor visual acuity.

## Background

Graves’ orbitopathy (GO) is an autoimmune disorder of the orbit, accompanied by a complex of ocular symptoms. It is the most common extrathyroidal manifestation of Graves’ disease (GD) and is associated with hyperthyroidism in 90% of cases [1]. GO primarily involves inflammatory changes in orbital tissues with potential thickening and fibrosis of the extraocular muscles (EOM) and orbital fat, increasing the volume within bony orbit [2]. The development of GO is characterized by progressive deterioration accompanying active symptoms, followed by a plateau, then gradual improvement toward the baseline [3]. Progressive deterioration is caused by an autoimmune process and characterized by lymphocyte infiltration, cytokine secretion, and fibroblast proliferation. In this active inflammatory phase, mainstay treatment includes systemic glucocorticoid therapy and orbital radiotherapy (RT) [4]. High-dose intravenous (IV) glucocorticoid is the first-line treatment for moderate-to-severe active GO patients, with studies reporting varying success rates from 54.3 to 77% [5–7], but relapse is frequent when they are withdrawn or tapered.

Orbital RT is used to improve symptoms during the progressive deterioration phase in GO patients and is particularly useful in treating those who are not sensitive to or tolerant of glucocorticoid therapy, or who have relapsing symptoms after glucocorticoid therapy [8]. Orbital RT is slower to show a therapeutic effect than glucocorticoid therapy but can be effective for a longer period [9]. Several retrospective studies have reported on the efficacy of orbital RT [10, 11], but prospective studies have yielded inconsistent results [12–14]. In randomized clinical trials, orbital RT was a safe treatment, but efficacy of RT was limited. The available data suggest particularly effective in EOM involvement, especially when it is of recent onset [15]. Since recent-onset active GO is much more treatable than longstanding or inactive disease, only certain patients can be expected to experience successful outcomes of orbital RT. Signs of inflammation, recent-onset eye muscle dysfunction, and optic neuropathy respond well to orbital RT, while proptosis and longstanding eye muscle restriction respond poorly [16]. However, the progression of GO varies among individual patients, so active GO is not always obvious in the early stages and is sometimes observed in long-lasting GO. There has been no study of orbital RT efficacy in long-lasting active GO.

We, therefore, aimed to evaluate the efficacy of orbital RT in patients with moderate-to-severe active GO including long-lasting disease and to determine factors that can predict treatment response.

## Methods

This study was approved by the institutional review board, and the requirement of informed consent was waived considering its retrospective design. This study adheres to the guidelines of the Declaration of Helsinki. Research data are stored in an institutional repository and will be shared upon request to the corresponding author.

We reviewed the medical records of 62 consecutive GO patients treated with RT between December 2015 and December 2018. Patients who were diagnosed with moderate-to-severe active GO, treated with RT, and followed up for at least 6 months were included in this study. These patients were refractory to high-dose IV steroids or were not eligible for treatment with high-dose IV steroids. The activity and severity of GO were accessed according to the standardized criteria recommended by the European Group on Graves’ Orbitopathy (EUGOGO) [17]. Patients who underwent ophthalmic surgery within three months prior to RT, pregnant patients, patients under 20 or over 80 years old, and patients with a history of other eye diseases such as glaucoma, diabetic retinopathy, or maculopathy, were excluded. Information on age, sex, duration of GD and GO prior to RT, smoking, family history of GO, and treatment history was collected. Serum concentrations of thyroid stimulating hormone (TSH), free thyroxine (FT4), and thyroxine binding inhibitory immunoglobulin (TBII) were evaluated prior to RT.

Comprehensive ophthalmologic examinations including the degree of proptosis, margin reflex distance 1 (MRD1, the vertical distance between the center of the pupil and the center of the upper eyelid), palpebral fissure height (PFH, the vertical distance between the center of the lower eyelid to the center of the upper eyelid), intraocular pressure (IOP), diplopia, EOM movement, visual acuity (VA), existence of compressive optic neuropathy (CON), and clinical activity score (CAS) were carried out before RT and at 3 and 6 months afterward. The degree of proptosis was measured using a Hertel exophthalmometer (Oculus, Arlington, VA, US). MRD1 and PFH were measured using custom-made PC-based EAS software (Eyelid Analysis Software, Biomedical Research Institute, Seoul, Korea) using photographs taken in primary position [18]. Diplopia was evaluated based on Gorman score [19]. EOM movement was measured using the Light Reflex Method, where a score of 45° was given when the light reflex was at the limbus, 30° at halfway between the limbus and pupil edge, and 15° at the pupil edge. Improvement in the EOM movement was defined if the variation was at least 15°, as previously described [20]. VA measured using a Snellen chart was converted to the logarithm of the minimum angle of resolution (logMAR). CON was judged based on the presence of decreased VA, plus one or more of the following findings: relative afferent pupillary defect, color vision deficit, or visual field defect [21]. A modified CAS was assessed using a seven-point modified formulation [21]. When differences in scores or measures existed for the two eyes of one patient, data from the worse eye were considered for analysis. All ophthalmic examinations were performed by one specialist (J.K.L).

Computed tomography (CT) was performed before and again at 3–6 months after RT at convenience. Orbital CT scans were obtained with 1 mm sections. Volumetric measurements of EOM and orbital fat were carried out as per Regensburg et al. [22] The volumes of the superior rectus, inferior rectus, medical rectus, lateral rectus, and orbital fat were calculated with the use of manual segmentation of CT scans with the Eclipse treatment planning system (TPS) (ver. 13.7, Varian Medical Systems Inc., Palo Alto, CA, USA). The orbital soft tissue CT numbers referenced for volume measurements were set at − 200 to − 30 Hounsfield units (HU) for fat tissue and − 30 to + 100 HU for muscle tissue. Tissues of interest with the chosen CT number in all slices were delineated and reconstructed in three dimensions and tissue volumes were measured.

All patients were treated with retroorbital irradiation using a linear accelerator with a three-dimensional conformal technique. Patients were immobilized with a custom-made thermoplastic case, and CT scanning with a slice thickness of 2.5 mm without contrast was performed for image acquisition and target contouring. The clinical target volume (CTV) included the EOMs and retroorbital fat. The lenses, optic nerves and lacrimal glands were zoned as organs-at-risk. A 2 mm margin around the CTV was generated as the planning target volume. The planned irradiation dose was 24 Gy in 12 fractions, and treatment planning was carried out using Eclipse TPS. Irradiation was performed using a 6 MeV photon beam. Concurrent low-dose oral steroids of 20 mg prednisone daily were administered to patients with active high CAS score for 2 ~ 4 weeks.

Response to orbital RT was defined as an improvement in at least two of the following six parameters: 1) CAS improvement in at least two points, 2) reduced proptosis by at least 2 mm, 3) improvement in diplopia (lessening of Gorman score), 4) improvement in EOM movement, 5) improvement in VA, and 6) disappearance of CON.

To verify the effects of timing of orbital RT on the treatment response, the subjects were divided into two groups based on the GO symptom duration from onset. The timing of RT according to GO duration was classified as the early-active phase until 24 months, and after 24 months as the late-active phase. Orbital RT in the late-active phase was performed in patients who recently experienced a distinct deterioration of symptoms or patients resistant to long-term steroid treatment. Response to orbital RT was analyzed and symptoms improved after RT was identified in each group.

All statistical analyses were performed using R version 3.4.0. Data are expressed as mean ± SD or median (interquartile range) for continuous variables. Categorical variables were reported as sample numbers and percentages. Changes in ophthalmic findings before and after treatment were compared using a linear mixed effect model or a generalized linear mixed effect model to analyze a fixed-time effect by controlling for random effects. For ordinal response variables, repeated measures proportional odds logistic regression was used. Changes in orbital fat and EOM volumes were compared using paired t-tests. Changes in ophthalmic findings according to the treatment phase were compared using the Wilcoxon signed-rank test or McNemar test. Univariable and multivariable logistic regression analyses were performed to analyze the effect of each clinical measurement on the binary response to RT. Variables showing significance levels of 0.1 in the univariable analysis were used in the multivariable analysis. All statistical analyses were considered significant at two-tailed *P* <  0.05.

## Results

### Patient characteristics

In total, 62 patients (23 male and 39 female) met the inclusion criteria. Their baseline characteristics are shown in Table 1. Their mean age was 52.08 ± 12.09 years. The median duration of GD was 43.7 (24.9–93.9) months and the median duration of GO was 25.5 (13.7–40) months. Forty-five patients (72.6%) had previously been treated with high-dose IV glucocorticoids. Low-dose oral steroids were administered to 34 patients (54.8%) during RT. Thirty patients continued to have active GO despite receiving high-dose IV steroid therapy and were administered supplemental steroids at low doses; four patients received low doses because they were not eligible for high-dose IV steroid therapy. Mean TSH and FT4 were within the normal range prior to RT. TBII was high at 15.02 ± 15.40 IU/L before RT and decreased to 2.56 ± 9.93 IU/mL within 6 months of RT.

**Table 1:** Baseline characteristics of patients

Characteristic	Value
Age, mean ± SD, years	52.08 ± 12.09
Sex, number (%)	
Male	23 (37.1%)
Female	39 (62.9%)
Duration of GD, median, months	43.7 (24.9–93.9)
Duration of GO, median, months	25.5 (13.7–40.0)
Smoking, number (%)	
Current smoker	11 (17.7%)
Ex-smoker	13 (20.9%)
Never smoked	38 (61.3%)
Family history of GO, number (%)	20 (32.3%)
Thyroid function, number (%)	
Hyperthyroidism	51 (82.3%)
Hypothyroidism	2 (3.2%)
Euthyroidism	9 (14.5%)
History of radioiodine therapy, number (%)	4 (6.5%)
History of thyroidectomy, number (%)	10 (16.1%)
Previous high-dose IV steroid use, number (%)	45 (72.6%)
Biochemistry	
TSH (0.55–4.78 μIU/mL), mean ± SD	1.68 ± 2.48
FT4 (0.89–1.76 ng/dL), mean ± SD	1.37 ± 0.63
TBII (0.01–1.75 IU/L), mean ± SD	15.02 ± 15.40
TBII 6 Months after radiotherapy, mean ± SD	2.56 ± 9.93

*SD* standard deviation, *GD* Graves’ disease, *GO* Graves orbitopathy, *RT* radiotherapy, *TSH* thyroid stimulating hormone, *FT4* free thyroxine, *TBII* thyroxine binding inhibitory immunoglobulin

### Response to orbital radiotherapy

**Fig. 1:**
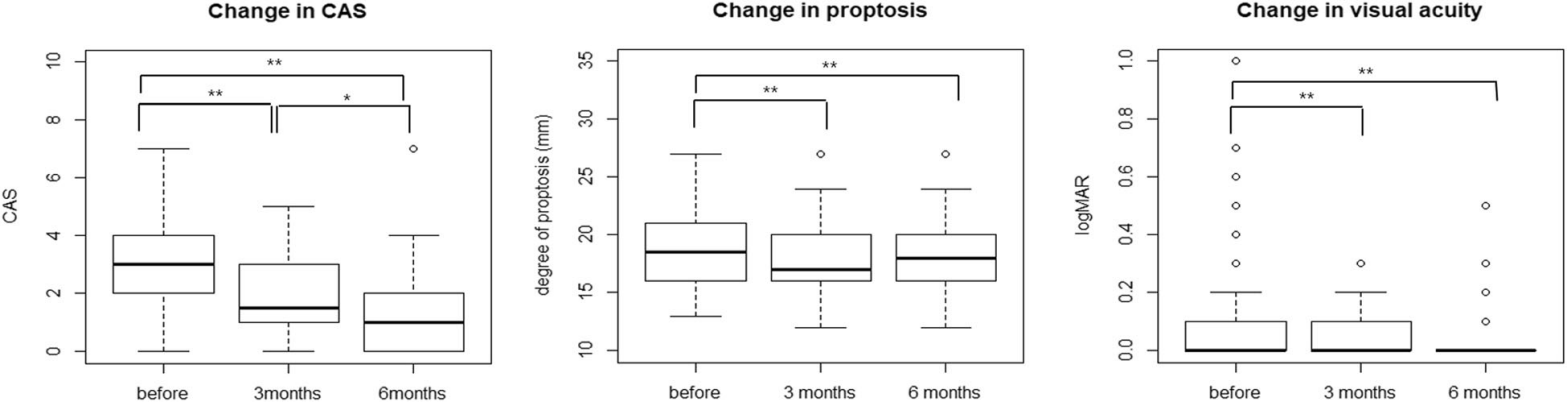
Changes in clinical activity score (CAS), proptosis, and visual acuity after radiotherapy. After orbital radiotherapy, CAS improved steadily, while proptosis and visual acuity improved for up to 3 months and then remained unchanged. **P <  0.01, *P <  0.05.

Forty (64.5%) and forty-six (74.1%) GO patients improved at 3 and 6 months after RT, respectively. Changes in individual ophthalmic findings after RT at these time points are shown in Table 2. CAS improved steadily, while proptosis and VA improved up to 3 months and then remained unchanged (Fig. 1). The number of patients with EOM movement ≤30° and CON also significantly decreased after RT. Diplopia improved after RT, but not significantly so. MRD1, PFH, and IOP were unchanged before and after RT. Volumes of the superior rectus, inferior rectus, medial rectus, lateral rectus, and orbital fat significantly decreased after RT (Table 3).

**Table 2:** Response to orbital radiotherapy

	Pre RT	3 months	6 months	*P* value
CAS	3.03 ± 1.87	1.81 ± 1.49	1.40 ± 1.44	< 0.001^a^
Degree of proptosis (mm)	18.66 ± 3.35	17.90 ± 3.17	17.82 ± 3.14	< 0.001^a^
MRD1 (mm)	4.08 ± 1.52	4.00 ± 1.41	3.79 ± 1.36	0.128^a^
PFH (mm)	9.61 ± 2.23	9.47 ± 2.05	9.31 ± 2.00	0.184^a^
Diplopia, Gorman score (number, %)				
Absent	9 (14.5)	9 (14.5)	13 (21.0)	0.157^b^
Intermittent	13 (21.0)	18 (29.0)	11 (17.7)	
Inconstant	20 (32.3)	18 (29.0)	22 (35.5)	
Constant	20 (32.3)	17 (27.4)	16 (25.8)	
EOM movement ≤30°, number of patients (%)	34 (54.84)	29 (46.77)	25(40.32)	0.012^c^
BCVA, logMAR	0.12 ± 0.20	0.04 ± 0.08	0.03 ± 0.08	< 0.001^a^
Number of CON (%)	13 (20.97)	4 (6.45)	3 (4.84)	0.008^c^
IOP (mmHg)	17.89 ± 3.26	17.82 ± 3.34	17.34 ± 3.65	0.127^a^

*CAS* clinical activity score, *MRD* margin reflex distance, *PFH* palpebral fissure height, *EOM* extraocular muscle, *VA* visual acuity, *CON* compressive optic neuropathy, *IOP* intraocular pressure, ^a^ linear mixed effect model, ^b^ repeated measure proportional odd logistic regression, ^c^ generalized linear mixed effect model

**Table 3:** Orbital volume change after radiotherapy

Orbital volume (cm^3^)	Before RT	After RT	P value^a^
Superior Rectus (cm^3^)	1.22 ± 0.50	1.08 ± 0.44	< 0.001
Inferior Rectus (cm^3^)	1.18 ± 0.50	1.09 ± 0.46	< 0.001
Medial Rectus (cm^3^)	1.45 ± 0.68	1.24 ± 0.56	< 0.001
Lateral Rectus (cm^3^)	1.24 ± 0.58	1.1.0 ± 0.46	< 0.001
Orbital fat (cm^3^)	23.95 ± 2.86	20.77 ± 2.72	< 0.001

^a^ Paired t-test

Complications included facial redness occurring in three patients (4.8%). This was well-controlled with the application of ointment. Cataract formation occurred in one patient (1.6%), who was treated with lens removal and intraocular lens implementation. Malignancy or radiation retinopathy was not detected.

### Factors predicting orbital radiotherapy response

Factors associated with a good response to RT in the univariable analysis included concurrent oral steroid use during RT (odds ratio [OR]: 3.75, 95% confidence interval [CI]: 1.11–12.65, *P* = 0.033); EOM limitation of < 30° (OR: 5.63, 95% CI: 1.56–20.31, *P* = 0.008); decreased VA (OR: 7.64, 95% CI: 1.56–37.47, *P* = 0.012); and the sum of EOM volume (OR: 1.18, 95% CI: 0.99–1.41, *P* = 0.050) (Table 4). GO duration was not prognostic for RT response. Multivariable analysis indicated that an EOM limitation of < 30° (OR: 5.13, 95% CI: 1.25–21.14, *P* = 0.043) and the sum of EOM volume (OR: 3.68, 95% CI: 1.22–11.11, *P* = 0.021) remained predictive of a good response to RT. Decreased VA tended to suggest a good response, but this did not reach statistical significance (OR: 4.32, 95% CI: 0.54–34.55, *P* = 0.168). Furthermore, concurrent oral steroid use during RT was not a significant factor in the multivariable analysis (OR: 0.99, 95% CI: 0.156–6.292, *P* = 0.921).

**Table 4:** Evaluation of predictive factors for the response of radiotherapy

Variable	Univariable		multivariable	
	OR (95% CI)	P value	OR (95% CI)	P value
Age (years)	1.02 (0.97–1.07)	0.497		
Sex (female)	1.02 (0.32–3.32)	0.969		
Duration of GO	1.01 (0.99–1.02	0.500		
Duration of GD	1.00 (1.00–1.01)	0.378		
Smoking		0.748		
Current smoker	1.0			
Ex-smoker	0.74 (0.10–5.49)			
Never-smoked	0.55 (0.10–2.94)			
Family history of GO (yes)	1.6 (0.44–5.78)	0.473		
Previous use of high-dose IV steroid (yes)	2.80 (0.83–9.40)	0.096	1.80 (0.208–15.484)	0.594
Concurrent oral steroid use during radiotherapy (yes)	3.75 (1.11–12.65)	0.033	0.99 (0.156–6.292	0.921
Radioiodine therapy (yes)	1.05 (0.10–10.84)	0.970		
Thyroidectomy (yes)	0.45 (0.11–1.86)	0.270		
Treatment phase (late)	0.55 (0.17–1.76)	0.317		
TSH (uIU/mL)	1.14 (0.83–1.57)	0.420		
free T4 (ng/dL)	0.69 (0.27–1.81)	0.459		
Initial TBII (IU/L)	1.02 (0.97–1.07)	0.403		
TBII change (IU/L)	0.99 (0.95–1.02)	0.966		
CAS	1.36 (0.96–1.92)	0.079	1.28 (0.76—2.14)	0.355
degree of proptosis (mm)	0.99 (0.83–1.17)	0.901		
Diplopia (Gorman score)		0.564		
absent	1.0			
Intermittent	2.67 (0.42–16.83)			
Inconstant	2.40 (0.46–12.61)			
constant	3.20 (0.58–17.72)			
EOM movement ≤30°	5.63 (1.56–20.31)	0.008	5.13 (1.25–21.14)	0.043
MRD1 (mm)	1.00 (0.69–1.46)	0.999		
PFH (mm)	1.02 (0.79–1.33)	0.863		
VA, logMAR	7.64 (1.56–37.47)	0.012	4.32 (0.54–34.55)	0.168
IOP (mmHg)	1.07 (0.89–1.29)	0.464		
Sum of EOMs (cm^3^)	1.18 (0.99–1.41)	0.050	3.68 (1.22–11.11)	0.021
Orbital fat (cm^3^)	1.16 (0.93–1.46)	0.193		

*OR odds ratio, CI* confidence interval, GO Graves’ orbitopathy, *GD* Graves’ disease, IV intravenous, RT radiotherapy, *TSH* thyroid stimulating hormone, *FT4* free thyroxine, *TBII* thyroxine binding inhibitory immunoglobulin, *CAS* clinical activity score, *EOM* extraocular muscle, *MRD* margin reflex distance, *PFH* palpebral fissure height, *VA* visual acuity, *IOP* intraocular pressure, *EOM* extraocular muscle

### Effect of the timing of orbital radiotherapy

Thirty patients received RT for early-active GO (duration ≤24 months) and 32 received RT for late-active GO (duration > 24 months). In patients receiving orbital RT in the late-active phase, the therapeutic goals were as follows: 1) reduction in CAS score (*n* = 13), 2) disappearance of CON (*n* = 6), and 3) improvement in EOM limitation or diplopia (n = 13). There were no significant differences in baseline demographics or biochemical characteristics between the early-active and late-active phase groups.

Six months after orbital RT, 22 (68.8%) patients in the late-active phase group and 24 (80.0%) patients in the early-active phase group showed improvement in GO. CAS improved in both groups, but proptosis improved only in the early phase group. Of patients with EOM movement ≤30°, 8 of 21 patients in the early phase group improved after RT, but only 1 in 13 patients in the late phase group improved. VA improved significantly in both groups, and the number of patients with CON decreased in both groups (Table 5).

**Table 5:** Comparison of outcome according to radiotherapy timing

	Early-active phase (*n* = 30)			Late-active phase (*n* = 32)		
	Pre RT	6 months	p	Pre RT	6 months	p
CAS	3.07 ± 1.74	1.23 ± 1.28	< 0.001^a^	3.00 ± 2.20	1.56 ± 1.58	< 0.001^a^
Proptosis (mm)	18.20 ± 3.39	17.10 ± 3.32	0.002^a^	19.00 ± 3.32	18.50 ± 2.83	0.061^a^
MRD1 (mm)	3.99 ± 1.43	3.73 ± 1.45	0.905^a^	4.15 ± 1.62	3.84 ± 1.30	0.199^a^
PFH (mm)	9.41 ± 2.25	9.06 ± 1.89	0.658^a^	9.80 ± 2.23	9.56 ± 2.10	0.407^b^
Diplopia, Gorman score (number, %)			0.819^a^			0.039^a^
Absent	3 (10.0)	4 (13.2)		6 (18.8)	9 (28.1)	
Intermittent	6 (20.0)	3 (10.0)		7 (21.9)	8 (25.0)	
Inconstant	9 (30.0)	12 (40.0)		11 (34.4)	10 (31.2)	
Constant	12 (40.0)	11 (36.7)		8 (25.0)	5 (15.6)	
EOM movement≤30°, number of patients (%)	21 (70.0)	13 (43.3)	0.013^b^	13 (40.6)	12 (37.5)	0.990^b^
VA, logMAR	0.12 ± 0.17	0.02 ± 0.05	0.006^a^	0.10 ± 0.23	0.04 ± 0.10	0.011^a^
Number of CON (%)	7 (23.3)	1 (3.3)	0.077^b^	6 (18.8)	2 (6.2)	0.134^b^
IOP (mmHg)	19.00 ± 3.26	17.80 ± 3.59	0.018^b^	16.80 ± 2.94	16.90 ± 3.71	0.468^a^

*CAS* clinical activity score, *MRD* margin reflex distance, *PFH* palpebral fissure height, *EOM* extraocular muscle, VA visual acuity, IOP intraocular pressure ^a^ Wilcoxon signed rank test, ^b^ McNemar test

## Discussion

Orbital RT has been used for GO patients as a treatment modality for around 60 years [23], but there have been conflicting reports on its effectiveness. Therefore, we attempted to assess the response rate for RT in patients with GO and tried to determine factors predicting treatment response. Our data demonstrate that 74.1% of patients with GO improved within 6 months of RT. This finding is comparable with responses to high-dose IV steroid treatment, which yields a response rate of 70–80% [24]. Although the patients in our study had limited conditions, which were refractory to high-dose IV steroids or were not eligible for treatment with high-dose IV steroids, our study confirmed that orbital RT is likely to be as effective as steroid therapy, which is the primary treatment for GO. Interestingly, only 64.5% of GO patients treated with RT showed an improvement in response after 3 months, lower than the response rate after 6 months. This indicates that there may be patients improving slowly even after 3 months post-RT, so it is necessary to take at least 6 months to judge the treatment outcome. The slow and long-lasting effect of RT may be a reason for the conflicting reports on the efficacy of RT.

Previous studies have reported that RT is more effective in treating EOM impairment and diplopia than in treating proptosis or soft tissue swelling [13, 15]. However, we found that orbital inflammation and proptosis, as well as EOM impairment and CON, improved after RT. Since orbital RT is recommended as second-line therapy in clinical practice, improvements in CAS and proptosis suggest that it may be an alternative to primary therapy instead of steroid treatment. The presence of fewer side effects in our study provided further support for the wider use of RT. Indeed, a recent study reported that a combination of high-dose IV steroid treatment and RT improved EOM limitation and decreased the reactivation of inflammation to a significantly greater extent than steroid treatment alone [25].

We found that ≤30° EOM limitation and larger EOM volume are good predictors of response to RT. During GO progression, soft tissue swelling and enlargement of EOM occur due to the inflammatory responses of orbital fibroblasts and lymphocytic infiltration. Our findings suggest that RT is effective for relieving edematous changes of EOM in patients with GO. Volumes of all EOM and orbital fat decreased significantly, and EOM limitation improved after RT. A previous study using MRI measurements also found that RT is effective in reducing the EOM volume in GO patients [26].

Several studies have reported that orbital RT is effective in restoring VA impairment and relieving CON [27]. We observed that 10 of 13 CON patients (76.9%) improved within 6 months of treatment and recovered to near-normal vision. Therefore, although our results did not indicate that decreased VA and CON were predictive of a good response to RT, RT should be considered as the preferred treatment modality in patients with CON. A favorable treatment outcome for patients with CON may be relevant to our findings that greater EOM volume predicts a good response to RT. All patients with CON also had enlarged EOM. In a multivariable analysis, variables strongly correlated with CON and VA impairment, such as EOM volume and EOM limitation, may cause an underestimation of the contributions of VA impairment and CON due to multicollinearity, despite statistical controls.

The efficacy of RT remains debatable, even in cases with prolonged GO. The natural course of GO is incompletely defined since it is impossible to determine in patients with moderate-to-severe GO who require immediate disease-modifying therapies [28]. Rundle’s curve, widely used as a descriptor of GO’s putative natural history, depicts progressive deterioration occurring over 6–24 months due to developing autoimmune processes [29]. Orbital RT can be expected to be effective only during the progressive deterioration phase since the underlying mechanism may be correlated with the inflammation process. With a longer GO period, inflammatory infiltration can be replaced by fibrosis, and thus, RT may be less effective. Several studies have emphasized that the selection of patients is important because patients with inactive GO are unlikely to respond to RT or glucocorticoids; thus, orbital RT is recommended in the early stages of the GO [15, 27]. A recent study also reported that a duration of symptoms longer than 18 months was significantly predictive of non-response to RT. [30] However, symptoms according to GO duration are very different in each patient. The active phase of GO can be prolonged or the disease can be reactivated by certain risk factors, including smoking [31]. In some patients, GO changes little in the early stages but may worsen during a prolonged GO period. These long-lasting active GO patients are not in the early phase but may remain in the progressive deterioration phase. Therefore, we separated active GO patients into two groups arbitrarily on the basis of a 24-month GO duration to verify the effect of RT according to duration. Of the patients included in our study, 32 remained in the late-active phase with a distinct deterioration of symptoms or resistant to long-term steroid therapy even after a GO duration of over 24 months. The improvement in CAS and VA after RT in these patients suggests that active treatment is still necessary to suppress inflammatory reactions after more than 24 months of GO duration. We found that a longer GO duration was not a poor prognostic factor. In contrast, only 1 of 13 patients with EOM limitation improved after RT. Fibrosis after prolonged inflammation may be responsible for a poor response to RT. Though diplopia seemed to improve after RT in these patients, the subjective nature of Gorman scoring for the assessment of diplopia may contradict the objective evaluation of ocular motility. In summary, irrespective of the length of GO duration, orbital RT in active GO patients with high CAS or visual disturbance may be a useful treatment for the relief of GO symptoms. Nevertheless, RT may not be effective in patients with an EOM limitation of ≤30° with more than 24 months’ GO duration.

Our study is limited by the relatively small number of patients localized to one tertiary hospital. Due to the nature of this hospital, some patients with a relatively longer GO duration due to previous treatment failures in other hospitals were also included in the study. There may also have been selection bias due to the nature of the retrospective observational study, which could have influenced the results. Prospective randomized studies are required to ascertain the effect of orbital RT on late-active GO.

## Conclusions

The efficacy of orbital RT was comparable with that of high-dose IV steroid therapy in treating GO patients who were refractory to high-dose IV steroids or who were not suitable for high-dose IV steroid treatment. In moderate-to-severe active GO patients, orbital RT may help improve high CAS, proptosis, EOM limitation, and CON. Enlargement of EOM and EOM limitation were predictive of a good response to RT. Orbital RT in long-lasting active GO patients may be a suitable therapy for the relief of symptoms including high CAS and poor VA.

## Data Availability

Research data are stored in an institutional repository and will be shared upon request to the corresponding author.

## Abbreviations

GO: Graves’ orbitopathy
GD: Graves’ disease
EOM: Extraocular muscles
RT: Radiotherapy
IV: Intravenous
EUGOGO: European Group on Graves’ Orbitopathy
TSH: Thyroid stimulating hormone
FT4: Free thyroxine
TBII: Thyroxine binding inhibitory immunoglobulin
MRD1: Margin reflex distance 1
PFH: Palpebral fissure height
IOP: Intraocular pressure
VA: Visual acuity
CON: Compressive optic neuropathy
CAS: Clinical activity score
HU: Hounsfield units
CTV: Clinical target volume
